# Critical Role of an MHC Class I-Like/Innate-Like T Cell Immune Surveillance System in Host Defense against Ranavirus (Frog Virus 3) Infection

**DOI:** 10.3390/v11040330

**Published:** 2019-04-06

**Authors:** Eva-Stina Isabella Edholm, Francisco De Jesús Andino, Jinyeong Yim, Katherine Woo, Jacques Robert

**Affiliations:** 1 Department of Microbiology and Immunology, University of Rochester Medical Center, Rochester, NY 14642, USA; eva-stina.i.edholm@uit.no (E.-S.I.E.); Francisco_Dejesus@URMC.Rochester.edu (F.D.J.A.); jyim3@u.rochester.edu (J.Y.); kwoo6@u.rochester.edu (K.W.); 2 The Norwegian College of Fishery Science, University of Tromsø, the Arctic university of Norway, 9037, Tromsø, Norway

**Keywords:** Unconventional T cell, nonclassical MHC, antiviral immunity, interferon

## Abstract

Besides the central role of classical Major Histocompatibility Complex (MHC) class Ia-restricted conventional Cluster of Differentiation 8 (CD8) T cells in antiviral host immune response, the amphibian *Xenopus laevis* critically rely on MHC class I-like (mhc1b10.1.L or XNC10)-restricted innate-like (i)T cells (iVα6 T cells) to control infection by the ranavirus Frog virus 3 (FV3). To complement and extend our previous reverse genetic studies showing that iVα6 T cells are required for tadpole survival, as well as for timely and effective adult viral clearance, we examined the conditions and kinetics of iVα6 T cell response against FV3. Using a FV3 knock-out (KO) growth-defective mutant, we found that upregulation of the XNC10 restricting class I-like gene and the rapid recruitment of iVα6 T cells depend on detectable viral replication and productive FV3 infection. In addition, by in vivo depletion with XNC10 tetramers, we demonstrated the direct antiviral effector function of iVα6 T cells. Notably, the transitory iVα6 T cell defect delayed innate interferon and cytokine gene response, resulting in long-lasting negative inability to control FV3 infection. These findings suggest that in *Xenopus* and likely other amphibians, an immune surveillance system based on the early activation of iT cells by non-polymorphic MHC class-I like molecules is important for efficient antiviral immune response.

## 1. Introduction

In mammals, potent adaptive immune response against viral pathogens largely depends on conventional Cluster of Differentiation (CD8) T cell effectors that express a broad T cell receptor (TCR) repertoire capable of recognizing a large array of antigens presented by polymorphic classical Major Histocompatibility Complex (MHC) class I molecules (class Ia). However, it is increasingly appreciated that other unconventional or innate-like T cell effectors are critically involved in antiviral immunity [1]. These iT cells interact with non-polymorphic MHC class I-like molecules, have a limited or invariant T Cell Receptor (TCR) repertoire, and are thought to serve as important early responders and immune regulators. One of the best characterized iT cell populations are invariant Natural Killer T (iNKT) cells, which are restricted by the MHC class I-like molecule Cluster of Differentiation CD1d that presents lipids [2,3]. The antiviral role of iNKT cells has been shown for murine cytomegalovirus (MCMV; [4]), lymphocytic choriomeningitis virus (LCMV; [5]), herpes simplex virus 1 (HSV1; [6]), HIV and influenza [7], and even for vaccinia virus (VAC; [8]). 

In the amphibian *Xenopus laevis*, while adult frogs exhibit an immune system whose conventional class Ia-restricted CD8 T cell compartment is dominant, as in mammals, tadpoles are immunocompetent but are naturally class Ia-deficient and primarily rely on MHC class I-like and iT cells to mediate immunity [9]. Deep-sequencing repertoire analysis has revealed the over-representation of 6 invariant TCRα rearrangements, implying the predominance of 6 larval iT cell subsets [10]. One of the 6 putative iT cell subsets, the iV6 T cell subset, expresses the rearranged Vα6-J1.43 TCRα chain and requires the MHC class I-like molecule XNC10 (encoded by mhc1b10.1.L) for its thymic development and function [10]. Notably, host immune responses against the ranavirus pathogen Frog Virus 3 (FV3) are significantly impaired by lack of iVα6 T cells resulting from XNC10 loss-of-function. FV3 is a major pathogen of amphibians, fish, and even reptiles [11]. The dramatic worldwide increases in host ranges (i.e., populations and species infected) and amphibian die-off caused by RVs (see eBook [12]) raise alarming concerns for biodiversity and aquaculture, and pose fundamental issues related to evolution of host/pathogen interactions. As such, the involvement of iT cells in addition to conventional CD8 T cells in antiviral host resistance is of high relevance.

In adult *X. laevis*, XNC10 deficiency considerably delays viral clearance, resulting in increased tissue damage [13]. As expected from iT cell prevalence in tadpoles, XNC10 deficiency markedly increases the mortality rate, especially at early stages of FV3 infection [10]. The generation of XNC10 tetramers by expressing XNC10 fused to *X. laevis* beta 2-microglobulin (b2m) has permitted a better characterization of the iVα6 T cell response kinetics and shows a rapid recruitment (within less than 24 h.) at the site of infection. This fast targeted response suggests a sensitive detection mechanism of FV3 infection. However, many facets of this response remain to be investigated, including whether iVα6 T cells require stimulation by XNC10 bound to viral or other ligands, or can be activated by XNC10-independent co-stimulation signals (e.g., danger signals, interferon [IFN] response). To explore some of these issues, we have taken advantage of our XNC10 tetramer, as well as a FV3 recombinant virus deficient for a putative immune evasion gene, to determine the role of productive versus ablated viral infection, as well as the effect of transiently impairing iVα6 T cell function at early stages of FV3 infection. 

## 2. Material and methods:

### 2.1. Animals 

All outbred *X. laevis* were from the *X. laevis* research resource for immunology at the University of Rochester (https://www.urmc.rochester.edu/microbiology-immunology/xenopus-laevis.aspx). All animals were handled in accordance with stringent laboratory and University Committee on Animal Research regulations, minimizing suffering (Approval number 100577/2003-151). For all experiments, three-week old tadpoles (stage 55, 1.5 cm long; [14]) and 1 year-old young adult frogs were used. 

### 2.2. Frog Virus 3 Stocks and Infection 

Baby hamster kidney cells (BHK-21, ATCC No. CCL-10) were maintained in Dulbecco's modified Eagle's medium DMEM (Invitrogen, Thermo Fisher Scientific, Inc. Waltham, MA, USA) containing 10% fetal bovine serum (Invitrogen), streptomycin (100μg/mL), and penicillin (100 U/mL) with 5% CO_2_ at 37 °C, then 30ºC for infection. The generation and characterization of the FV3 knock-out (KO) mutant ∆vCARD (or ∆64R) has been detailed elsewhere [15,16,17]. Wild type (WT) and KO FV3 were grown using a single passage through BHK-21 cells and were subsequently purified by ultracentrifugation on a 30% sucrose cushion. Adult frogs were infected by intraperitoneal (i.p.) injection of 1 × 10^6^ PFUs in 100 µL of Amphibian Phosphate buffered saline (APBS) and tadpoles were infected by i.p. injection of 10,000 PFUs in 5 µL APBS. Uninfected control animals were mock-infected with an equivalent volume of APBS. At different days post-infection (dpi), animals were euthanized using 1 µg/L tricaine methanesulfonate (TMS) buffered with bicarbonate prior to dissection and extraction of nucleic acids from tissues.

### 2.3. Quantitative Gene Expression Analyses

Total RNA was extracted from peritoneal leukocytes and kidneys using Trizol reagent, following the manufacturer's protocol (Invitrogen). The cDNA was synthetized with 0.5 µg of RNA in 20 µL using the iScript cDNA synthesis kit (Bio-Rad, Hercules, CA, USA), and 1 µL of cDNA template was used in all RT-PCRs and 150 ng DNA for PCR. Minus RT controls were included for every primer pair. A water-only control was included in each reaction. The qPCR analysis was performed using the ABI 7300 real-time PCR system with PerfeCT SYBR Green FastMix, ROX (Quanta, Beverly, MA, USA), and ABI sequence detection system (SDS) software. Glyceraldehyde-3-phosphate dehydrogenase (GAPDH) controls were used in conjunction with the ∆∆ CT method to analyze cDNA for gene expression. All primer sequences are listed in Table S1.

### 2.4. Viral Load Quantification by qPCR and Plaque Assay

FV3 viral loads were assessed by absolute qPCR by analysis of isolated DNA in comparison to a serially diluted standard curve. Briefly, an FV3 DNA Pol II PCR fragment was cloned into the pGEM-T Easy vector (Promega, Madison, WI, USA). This construct was amplified in bacteria, quantified, and serially diluted to yield 1010-101 plasmid copies of the FV3 DNA polymerase II. These dilutions were employed as a standard curve in subsequent absolute qPCR experiments to derive the viral genome transcript copy numbers relative to this standard curve. Virus quantification by plaque assay was performed on BHK-21 monolayers in 6-well plates under an overlay of 1% methylcellulose [18]. Infected cells were cultured for 7 days at 30 °C in 5% CO_2_. Overlay media was aspirated and cells were stained for 10 min with 1% crystal violet in 20% ethanol.

### 2.5. XNC10 Tetramer Production

XNC10 tetramers were generated as previously described [10]. Briefly, beta 2 microglobulin (b2m) was linked via a 23-aa Glycine rich C-terminal flexible linker to the α1-α3 domains of XNC10 containing a BirA site-specific biotinylation site at the end of the α3 domain and cloned into the pMIBV5-HisA expression vector (Invitrogen). The b2m-linker-XNC10 construct was expressed in Sf9 insect cells and monomeric b2m-linker-XNC10 was purified by Ni-NTA-Agarose Chromatography (Qiagen, Hilden, Germany) and concentrated to 1 μg/μL using Amicon Ultra Centrifugal Filter (Millipore, Burlington, MA, USA). BirA enzymatic biotinylation was performed for 18 h. at 30 °C according to the manufacturer’s protocol (Avidity, Aurora, CO, USA), and the purified biotinylated proteins were extensively dialyzed against APBS, pH 7.5, to remove any unbound biotin. XNC10 tetramers were generated by incubating b2m-linker-XNC10 with fluorochrome-labeled streptavidin at a 5:1 ratio at room temperature for 4 h. before use. Purified XNC10 tetramers (1 μg) were injected intra i.p. at a volume of 5 μL.

### 2.6. Statistical Analysis

One way ANOVA followed by Dunn’s or Tukey’s multiple comparison tests were used for statistical analysis of expression and viral load data. Analyses were performed using a Vassar Stat online resource (http://vassarstats.net/utest.html). Statistical analysis of survival data was performed using a Log-Rank Test (GraphPad Prism 6, San Diego, CA, USA). A probability value of *p* < 0.05 was used in all analyses to indicate significance. Error bars on all graphs represent the standard error of the mean (SEM).

## 3. Results

### 3.1. Relationships Between FV3 Infection Magnitude and iVα6 T cell Response in Adult X. Laevis

**Figure 1:**
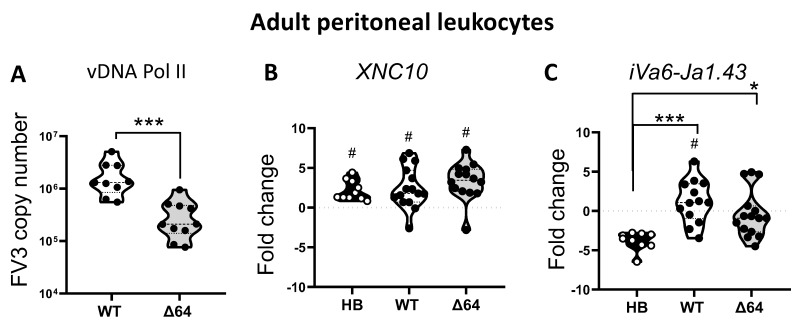
Effects of infection with attenuated knockout (KO) FV3 recombinant or bacterial stimulation on iVα6 T cell response. Peritoneal leukocytes (PLs) were collected at 1 dpi from adult frogs infected with 1 × 10^6^ PFUs of WT-FV3 or ∆64-FV3, or 100 µl heat-killed (HB) *E. coli*. (**A**) Genome copy number using absolute qPCR with primers against FV3 DNA polymerase II. (**B**) XNC10 relative gene expression and (**C**) iVα6-Jα1.43 relative gene expression. Gene expression was determined relative to an endogenous control (GAPDH) and fold changes were calculated using the unstimulated sample (injected with equivalent volume of APBS) collected at the same time point. Data are pooled from three independent experiments with *n* = 4–5 animals in each experiment and each dot represents an individual animal. The line intersecting the *y*-axis at 0 represents the unstimulated control that the fold changes of the treatments are in relation to; (#) *p* < 0.05 significant differences compared to unchallenged (APBS) injected controls; (*) *p* < 0.05 and (***) *p* < 0.001 statistically significant differences between the indicated groups (one way ANOVA and Dunns’s multiple comparison test).

We have previously demonstrated that FV3 infection in adult *X. laevis* elicits a transitory influx of the innate (i)T cell subset iVα6 T into the peritoneal cavity [13]. To delineate the factors governing iVα6 T cell recruitment during a FV3 infection, we took advantage of the FV3 KO mutant ∆vCARD (64R)-FV3 that has previously been shown to have attenuated virulence and growth in vivo, eliciting different host responses [14,17]. Accordingly, we i.p infected adult *X. laevis* with wild type (WT) FV3 or ∆64-FV3 for 24 h. before collecting peritoneal leukocytes (PLs) and quantified transcript levels of the specific invariant Vα6-Jα1.43 rearrangement. As a control we also challenged frogs with heat-killed *E. coli*. This type of bacterial stimulation has been shown to induce a strong nonspecific inflammatory response but does not stimulate the recruitment or accumulation of iVα6 T cells in the peritoneal cavity [13]. Because iVα6 T cells interact with the MHC class I-like molecule XNC10, we also examined its gene expression profile. To evaluate viral loads, we determined the FV3 genome copy number using absolute qPCR. Consistent with previous findings, ∆64-FV3 infection resulted in significantly (*p* = 0.005) lower viral load compared to WT-FV3 already at 1 dpi [17]. All treatments resulted in a significantly increased XNC10 expression compared to PLs collected from APBS-injected control frogs at the same time point, with no difference among the treatment groups (Figure 1B). However, only WT-FV3 infection resulted in significantly elevated iVα6-Jα1.43 transcript levels compared to uninfected controls (*p* = 0.018), whereas in frogs injected with heat-killed bacteria, iVα6-Jα1.43 expression was lower compared to controls, presumably due to the large influx of immune cells into the peritoneal cavity. Although the high individual variation prevented statistical significance among the two FV3 infected groups, iVα6-Jα1.43 transcript levels observed with ∆64-FV3 mutants were elevated above sham-infect APBS controls in only 20% of frogs (3 out of 15 frogs) compared to 70% (9 out of 13) for WT-FV3 infected frogs, suggesting a correlation between iVα6 recruitment and the level of viral replication.

**Figure 2:**
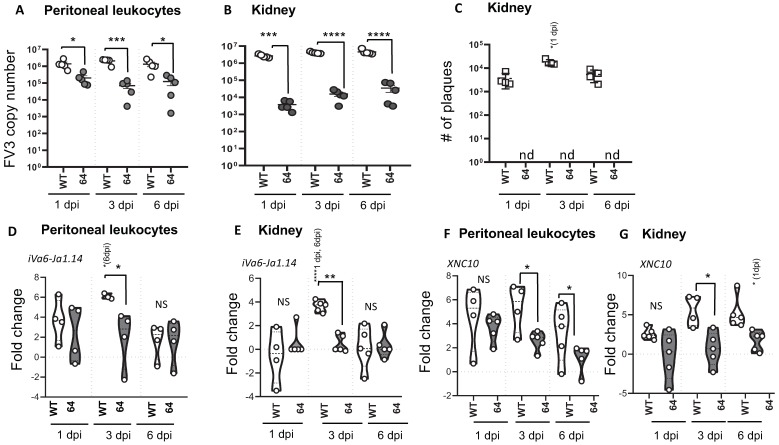
Magnitude of iVα6 T cell response in adult frogs is associated to the level of viral replication and production of infectious particles. PLs and kidneys were collected at 1, 3, and 6 dpi from adult frogs infected with 1 × 10^6^ PFUs of WT-or ∆64-FV3. FV3 genome copy number by absolute qPCR in PLs (**A**) and kidneys (**B**) were determined. The total number of infectious particles (nd: not detected) in the kidney was determined by plaque assay (**C**).Gene expression of iVα6-Jα1.43 and XNC10 in PLs (**D**,**F**) and kidneys (**E**,**G**) were determined relative to an endogenous control (GAPDH) and fold changes were calculated using mock-infected frogs as a control. Each dot represents an individual animal (*n* = 4–5). The line intersecting the *y*-axis at 0 represents the APBS control that the fold changes of the treatments are in relation to. Note: * *p* < 0,05; ** *p* < 0.01; *** *p* < 0.001, and **** *p* < 0.0001 above the line denotes statistically significant differences between the different treatment groups; significant differences between time points within each treatment group are indicated within parentheses; NS indicates no significant differences (one way ANOVA and Dunns’s multiple comparison test).

To obtain additional evidence of the correlation between active viral replication and iVα6 T cell recruitment, we i.p. infected adult *X. laevis* with WT- and ∆64-FV3, and then monitored the transcript levels of iVα6-Jα1.43 and XNC10 in the peritoneal cavity (the site of infection) and kidney (main site for viral replication) at 1, 3, and 6 dpi (Figure 2). To evaluate viral replication, we determined the genome copy number by absolute qPCR (Figure 2A,B) and to assess productive infection we performed plaque assays (Figure 2C and Figure S1). Again ∆64-FV3 exhibited a severe replication defect preventing the production of infectious particles (Figure 2C and Figure S1). Using iVα6-Jα1.43 expression as a proxy for monitoring the kinetics of iVα6 T cell recruitment, we detected an increase in iVα6-Jα1.43 transcript levels in the peritoneal cavity as early as 1 dpi with WT-FV3 compared to uninfected controls, which became significant by 3 dpi, and then returned to low but detectable levels by 6 dpi (Figure 2D). In contrast, ∆64-FV3 infection did not trigger a significant increase in iVα6-Jα1.43 transcript levels. In kidneys, iVα6-Jα1.43 expression was significantly elevated at 3 dpi and then returned to baseline at 6 dpi following infection with WT-FV3 (Figure 2E). In contrast, no significant changes in iVα6-Jα1.43 expression were detected with ∆64-FV3 infection.

The examination of the expression response of the iVα6 T cell restricting the MHC class I-like XNC10 gene revealed some interesting correlations. In PLs, WT-FV3 infection induced a rapid (from 1 dpi) and sustained (until 6 dpi) XNC10 increase (Figure 2F), which was delayed (from 3 to 6 dpi) in kidneys (Figure 2G). Consistent with the poorly induced iVα6-Jα1.43 expression, infection with ∆64-FV3 also resulted in impaired induction of XNC10 gene expression. Indeed, ∆64-FV3 infection did not induce any significant change in XNC10 gene expression at 3 and 6 dpi in both PLs and kidneys.

Collectively, these data indicate that XNC10-restricted iVα6 T cell recruitment is relative to viral burden and requires active viral replication.

### 3.2. Relationships Between FV3 Infection Magnitude and iVα6 T Cell Response in Tadpoles

**Figure 3:**
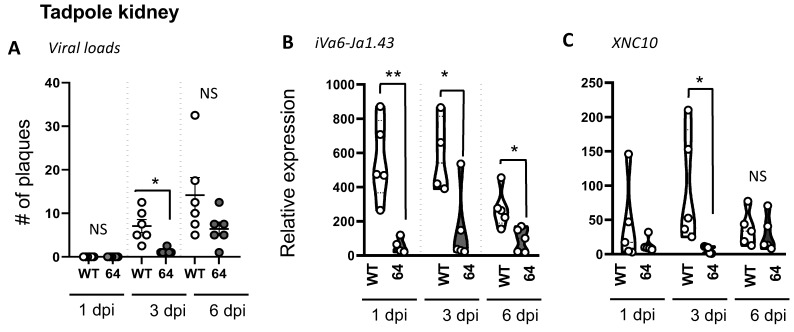
The iVα6 T cell response in tadpoles depends on active viral replication and productive FV3 infection. Three week-old (stage 55) tadpoles were infected with 10,000 PFUs of WT-or ∆64-FV3. At 1, 3, and 6 dpi, kidneys were collected and the total number of infectious particles was determined by plaque assay, respectively (**A**). Gene expression of iVα6-Jα1.43 (**B**) and XNC10 (**C**) was determined relative to an endogenous control (GAPDH) and relative expression was calculated against the lowest observed expression according to the ∆∆Ct method (*n* = 5). No iVα6-Jα1.43 transcripts were detected in the kidney uninfected tadpoles. * *p* < 0.05 and ** *p* < 0.01 above the line denotes statistically significant differences between treatment groups; NS indicates no significant differences (one way ANOVA and Dunn’s multiple comparison test).

In contrast to adult frogs that exhibit a prominent conventional antiviral CD8 T cell response restricted by classical MHC class Ia, tadpoles have barely detectable class Ia surface protein expression and rely heavily on XNC10-restricted iVα6 T cells [10]. Indeed, XNC10-deficient transgenic tadpoles lacking iVα6 T cells succumb to FV3 infection much faster than controls. This suggests that a rapid iVα6 T cell response is required during the early stage of FV3 infection. Similar to adult frogs, we postulated that the efficient recruitment of iVα6 T cells at the site of infection in tadpoles would depend on a productive FV3 infection. Accordingly, we i.p. infected tadpoles with either WT- or ∆64-FV3. Consistent with previous reports, Δ64-FV3 exhibited a severe growth defect in tadpoles, as shown by the very low number of plaques detected, even at 6 dpi (Figure 3A and Figure S2). As expected, WT-FV3 infection elicited an iVα6 T cell response that was overall comparable to adults, including an early (by 1 dpi) increase in iVα6-Jα1.43, as well as XNC10 transcript levels that were maintained by 3 dpi and then declined close to background levels by 6 dpi. In contrast, there were no significant changes in XNC10 and iVα6-Jα1.43 transcript levels following ∆64-FV3 infection (Figure 3B,C).

### 3.3. Targeted iVα6T cell depletion in vivo

**Figure 4:**
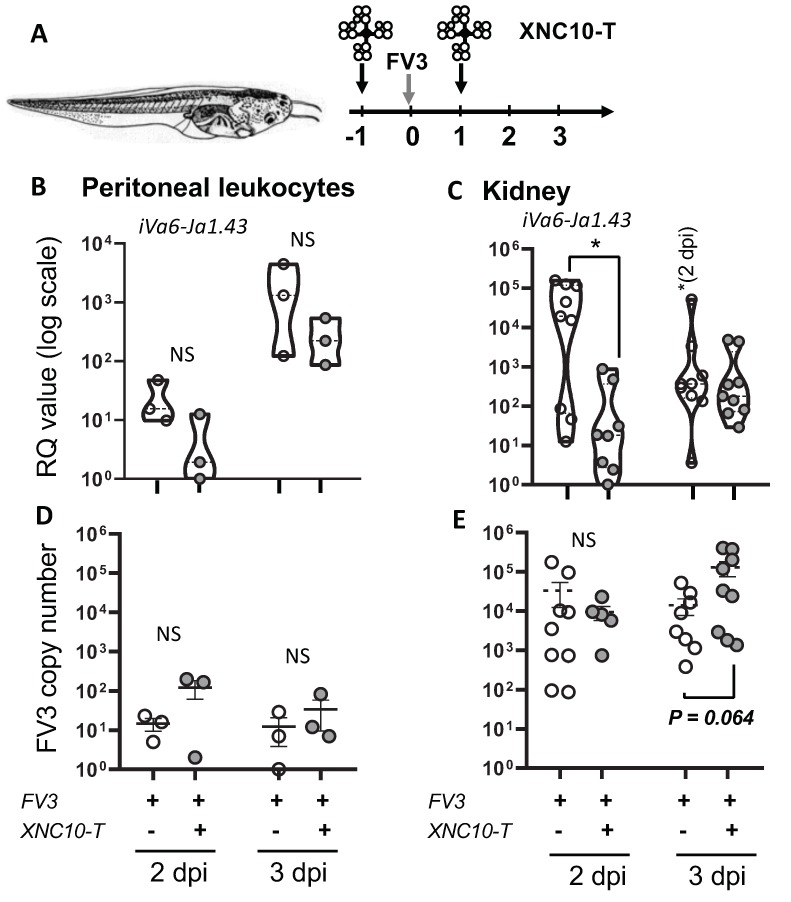
XNC10 tetramer-mediated iVα6T cell depletion in tadpoles affects iVα6-Jα1.43 transcript levels and viral replication. PLs and kidneys were collected from three week-old (stage 55) tadpoles that had been injected with 1 μg XNC10 tetramers (XNC10-T) or vehicle control 1 day pre, and 1 day post i.p. injected with 10,000 PFUs of FV3, at the indicated time points (*n* = 9). A schematic of the injection regime is shown in (**A**). Gene expression of iVα6-Jα1.43 in PLs (**B**) and kidneys (**C**) is shown. Results are normalized to an endogenous control and presented as relative expression compared with the lowest observed value according to the ∆∆Ct method. FV3 loads in PLs (**D**) and kidneys (**E**) were measured using absolute qPCR with primers against FV3 polymerase II. For PLs, each dot represents a pool of 3 tadpoles, while for kidneys, each dot represents a single tadpole; * *p* < 0.05 denotes statistically significant differences between the indicated groups; NS indicates no significant differences (One way ANOVA followed by Tukey’s multiple comparison test).

As mentioned, intraperitoneal (i.p.) infection of *X. laevis* tadpoles with FV3 results in a rapid and transitory increase in Vα6-Jα1.43 transcript levels, both at the site of infection and in the kidney (main FV3 target organ), consistent with a direct and active involvement of this specific iT cell population in the early anti-FV3 response [13]. In support of this possibility, the developmental disruption of iVα6 T cells in MHC class I-like XNC10-deficient transgenic tadpoles results in increased susceptibility to FV3 [10]. However, silencing of XNC10 from early embryogenesis is likely to result in biological effects other than those inferred via the reciprocal loss of a unique iT cell population. Therefore, to more directly determine the role of iVα6 T cells in anti-viral defense against FV3 in the presence of a functional XNC10 gene, we targeted iVα6 T cells using XNC10 tetramers. XNC10 tetramers have been shown to selectively induce iVα6T cell death ex vivo and to temporarily ablate Vα6-Jα1.43 transcript levels in vivo (Banach et al., submitted). Accordingly, we injected *X. laevis* tadpoles with 1 μg of XNC10 tetramers 1 day prior and 1 day following i.p. injection with FV3 and quantified transcript levels of the invariant Vα6-Jα1.43 rearrangement in PLs and kidneys at different times following infection (Figure 4). Control groups received injections with an equivalent volume of amphibian PBS at the same time points. To evaluate viral loads and dissemination in the different groups, we determined the FV3 genome copy number using absolute qPCR (Figure 4D,E). In addition, we examined the gene expression of XNC10 to evaluate any impact of the injection regime on the expression of this MHC class I-like gene (Figure S3).

Consistent with XNC10 tetramers inducing iVα6 T cell death, iVα6-Jα1.43 transcripts were markedly reduced, albeit not significantly, in the peritoneal cavity (site of infection) at 2 dpi, and remained lower than controls at 3 dpi. In the kidney, iVα6-Jα1.43 transcripts were significantly lower in the XNC10 tetramer treated group at 2 dpi, reaching levels equivalent to controls by 3 dpi. It is noteworthy that consistent with previous reports, iVα6-Jα1.43 transcripts were either undetected or near threshold levels in uninfected tadpoles. Thus, XNC10 tetramer treatment provides an efficient transient depletion of iVα6 T cells in vivo, providing us with a system to directly investigate the functional roles of iVα6 T cells. No significant difference was observed in XNC10 expression between the two groups (Figure S3).

**Figure 5:**
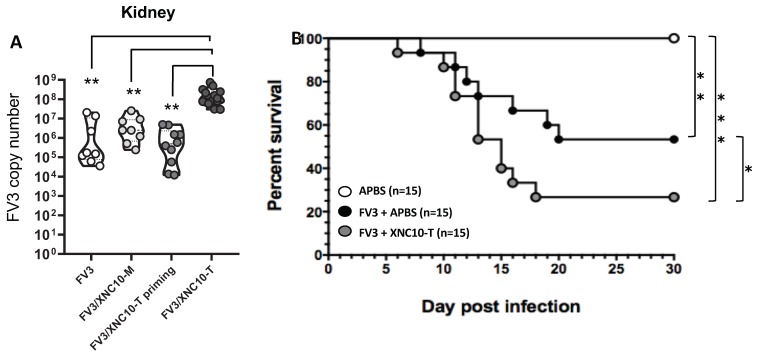
Specificity and long term impact of transitory iVα6T cell depletion. (**A**) Three week-old (stage 55) tadpoles were injected with either 1 μg XNC10-tetramers (FV3/XNC10-T), 1 μg XNC10-monomers (FV3/XNC10-M), or vehicle control (FV3/APBS) 1 day pre and 1 day post i.p. infection with 10,000 PFUs of FV3 (*n* = 8), and kidneys were collected at 6 dpi. The last group (FV3/XNC10-T priming) was first injected with 1 μg XNC10-tetramers, then 3 days later infected with FV3, and kidneys were collected at 6 dpi. Viral loads were assessed by absolute qPCR with primers against FV3 polymerase II. The results are combined from two separate experiments, and each dot represents an individual tadpole; ** *p* < 0.01 above the line denotes statistically significant differences between the indicated groups (One way ANOVA followed by Tukey’s multiple comparison test). (**B**) Three week-old (stage 55) tadpoles were injected with either 1 μg XNC10 tetramers (FV3/XNC10-T) or vehicle control (FV3/APBS) 1 day pre, and 1 day post were i.p injected with 10,000 PFUs of FV3 (*n* = 15) and survival was monitored daily over a 30-day period. Survival was determined using Kaplan-Meier, * *p* < 0.05, ** *p* < 0.005, and *** *p* < 0.0005. Uninfected controls (white circle), FV3 infected tadpoles (black circle), and XNC10 tetramer treated FV3 infected tadpoles (grey circle).

**Figure 6:**
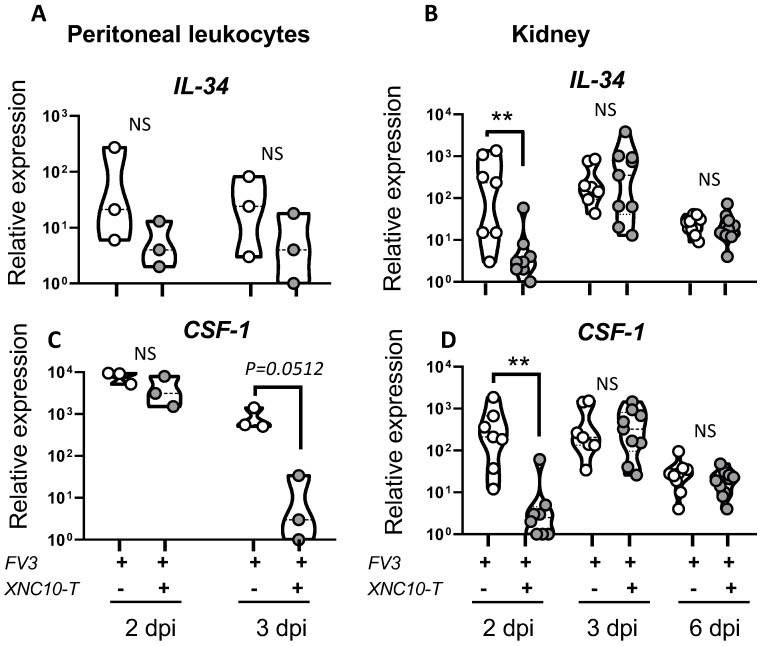
Effect of XNC10 tetramer treatment on the expression of the macrophage stimulating factor genes CSF-1 and IL-34 in the peritoneal cavity and kidneys of FV3 infected tadpoles. PLs and kidneys were collected from three week-old (stage 55) tadpoles that had been injected with 1 μg XNC10 tetramers or vehicle control 1 day pre- and 1 day post-i.p. injection with 10,000 PFUs of FV3, at the indicated time points (*n* = 8–9). Quantitative gene expression analysis of IL-34 (**A**,**B**) and CSF-1(**C**,**D**) were determined relative to a endogenous control (GAPDH), and relative expression was calculated against the lowest observed expression according to the ∆∆Ct method (*n* = 9); ** *p* < 0.005 above the line denotes statistically significant differences between the different treatment groups; NS indicates no significant differences (One way ANOVA followed by Tukey’s multiple comparison test).

In regard to viral loads, while there was no significant difference in viral genome copy numbers in the peritoneal cavity, XNC10 tetramer treated tadpoles displayed a significantly (*p* = 0.005) higher viral load in the kidneys by 3 dpi (Figure 5B). By 6 dpi, using the same injection scheme as described in Figure 4A, there were significantly higher viral loads in the kidneys of the XNC10-tetramer treated group compared to vehicle control (Figure 5A). Notably, viral loads were not increased in tadpoles injected with XNC10 monomers, which do not bind to iVα6 T cells [10]. In addition, XNC10 tetramer treatment had only a transitory effect on iVα6 T antiviral activity, as shown by comparable viral loads detected when FV3 infection occurred 3 days rather than 1 day after XNC10 tetramer injection (Figure 5A). To assess whether this increase in viral loads had an impact on host resistance, we determined the cumulative mortality and mean survival of XNC10 tetramer injected and FV3 challenged tadpoles (Figure 6). Notably, transitory depletion of antiviral iVα6 T cells within 24 h. of FV3 infection resulted in increased mortality rate compared with FV3 infected controls (*p* = 0.05). The seven-fold increase in post-mortem viral loads found in infected XNC10 tetramer-treated tadpoles that died within 6 to 20 dpi compared to vehicle treated infected controls (Table S2) further suggests that the increased mortality was due to an inefficient control of viral infection. These data support the conclusion that iVα6 T cells are intricately involved in *X. laevis* anti-FV3 immune responses and highlight the importance of an efficient onset of appropriate anti-viral immune response in order to control and efficiently combat FV3 infection.

### 3.4. Effects of iVα6T cell depletion on PLs and kidney antiviral responses in tadpoles

We have previously shown that the compromised anti-FV3 response observed in XNC10-deficient transgenic frogs correlates with the induction of macrophages, exhibiting a less potent antiviral state [10]. In particular, XNC10 deficiency hampered the expression of the macrophage growth factor IL-34. Notably, IL-34 has been shown to elicit the differentiation of mononuclear phagocytes into robust type I interferon (type I IFN), producing macrophages exhibiting strong FV3 antiviral activity [19]. These findings prompted us to hypothesize that iVα6 T cells promote timely and efficient type I IFN-mediated anti-FV3 responses by influencing the polarization of macrophages into a more potent antiviral state. Thus, to further delineate the putative functional roles of iVα6 T cells during FV3 infection, we next determined if direct and transient iVα6 T cell depletion has an impact on macrophage effector functions. To address this possibility, we monitored the expression profiles of the two macrophage growth factors CSF-1 [20] and IL-34 [19] in PLs and kidneys (Figure 6) from FV3 infected tadpoles, with or without XNC10 tetramer treatment. Both IL-34 and CSF-1 transcription levels were significantly lower in the kidneys of XNC10 tetramer treated tadpoles at 2 dpi, correlating with the reduced iVα6-Jα1.43 expression, and levels comparable to controls were reached by 3 dpi.

**Figure 7:**
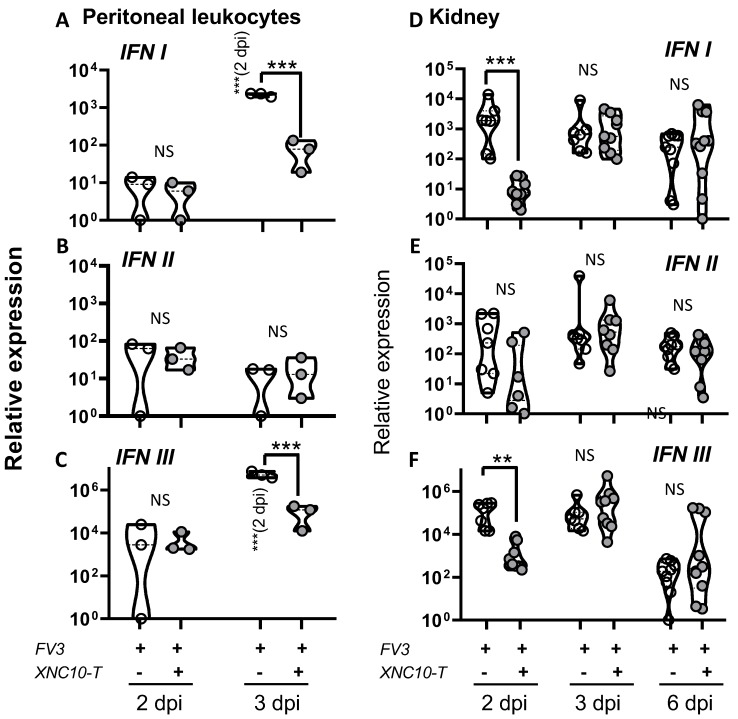
XNC10 tetramer treatment results in a delayed antiviral response in the peritoneal cavity and kidneys of FV3 infected tadpoles. PLs and kidneys were collected from three week-old (stage 55) tadpoles that had been injected with 1 μg XNC10 tetramers (XNC10-T) or vehicle control 1 day pre- and 1 day post-i.p. injection with 10,000 PFUs of FV3, at the indicated time points (*n* = 8–9). Quantitative gene expression analysis of type I (**A**,**D**), type II (**B**,**E**), and type III IFN (**C**,**F**) were determined relative to a endogenous control (GAPDH), and relative expression was calculated against the lowest observed expression according to the ∆∆Ct method (*n* = 9); * *p* < 0.05, ** *p* < 0.005, and *** *p* < 0.001 above the line denote statistically significant differences between the different treatment groups; significant differences between time points within each treatment group is indicated within parentheses; NS indicates no significant differences (One way ANOVA followed by Tukey’s multiple comparison test).

Given that the type I IFN response is critical in controlling viral infections and is compromised in XNC10-deficient animals [13], we postulated that a direct impairment of iVα6 T cells should affect type I IFN gene expression to a similar extent. Indeed, unlike control animals, both type I IFN and type III IFN gene expression were magnitudes lower at 3 dpi in PLs of XNC10 tetramer treated animals compared to controls, whereas the levels of type II IFN were highly variable between animals, with no apparent difference in expression between the two groups (Figure 7A–C). Similarly, in kidneys the expression of both type I and type III IFN genes was significantly impaired in XNC10 tetramer treated animals at 2 dpi, whereas by 3 dpi both treated and control groups exhibited high transcript levels of type I and type III IFN (Figure 7D,E).

**Figure 8:**
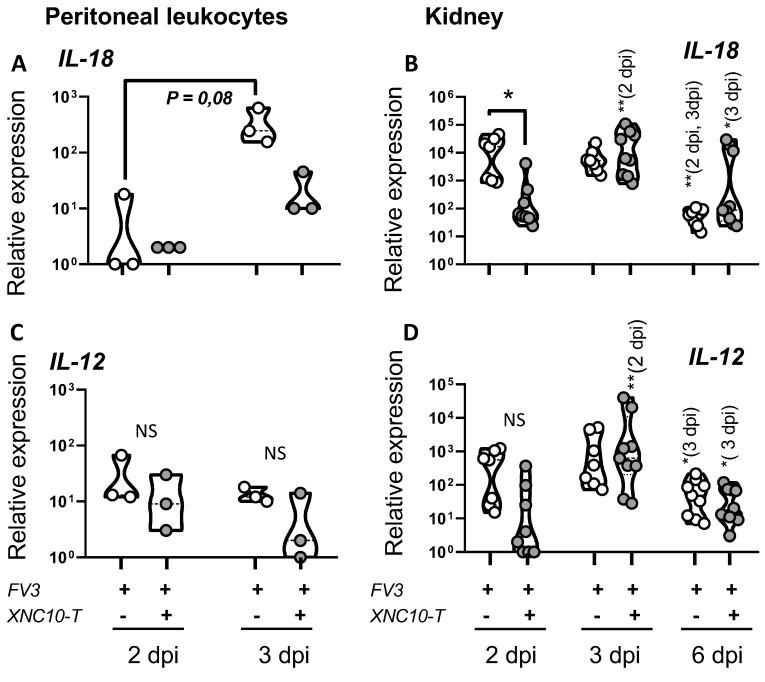
Effects of iVα6T cell depletion on IL-18 and IL-12 responses. PLs and kidneys were collected from three week-old (stage 55) tadpoles that had been injected with 1 μg XNC10 tetramers (XNC10-T) or vehicle control 1 day pre- and 1 day post-i.p. injection with 10,000 PFUs of FV3, at the indicated time points (*n* = 8–9). Quantitative gene expression of IL-18 (**A**,**B**) and type IL-12 (**C**,**D**) was determined relative to an endogenous control (GAPDH) and relative expression was calculated against the lowest observed expression according to the ∆∆Ct method (*n* = 9); * *p* < 0.05; ** *p* < 0.005, above the line denotes statistically significant differences between the different treatment groups; significant differences between time points within each treatment group is indicated within parentheses; NS indicates no significant differences (One way ANOVA followed by Tukey’s multiple comparisons test).

In response to a viral infection, protective host response relies on the onset of appropriate cell-mediated immunity to mediate viral clearance. Thus, we next examined the expression of interleukin18 (IL-18) and interleukin 12 (IL-12) upon FV3 infection in PLs and kidneys of control and iVα6 T cell deficient tadpoles. Both IL-18 and IL-12 are produced by macrophages and act in synergy to promote the development of innate and specific Th1 type immune responses [21,22,23]. Given that iVα6 T cell deficiency hampers the induction of macrophage differentiating factors, resulting in a delayed interferon response, we postulated that IL-18 and IL-12 cytokine production would be similarly impaired. Indeed, unlike control animals, IL-18 expression was significantly lower at 2 dpi in kidneys of XNC10 tetramer treated animals, whereas at 3 dpi XNC10 tetramer treated tadpoles exhibited elevated IL-18 transcript levels on par with that of controls that were significantly reduced by 6 dpi in both groups (Figure 8). While there was a slight decrease in IL-12 gene expression at 2 dpi in XNC10 tetramer treated animals, it was not statistically significant.

Overall, these findings are in accordance with what had previously been reported for XNC10-deficient transgenic animals (that lack both XNC10 and iVα6 T cells, [13]), suggesting that iVα6 T cells are directly and critically involved in mounting an anti-FV3 response, and that acute loss or impairment of these cells result in both a delayed type I and type III IFN antiviral response, as well as a delayed IL-18 response.

## 4. Discussion

The present study provides new insights into the requirement for an effective amphibian host response to the ranavirus FV3 trough, an early detection by the MHC class I-like XNC10, and the rapid recruitment of the activated XNC10-restricted innate T cell subset iVα6. A remarkable feature of this novel antiviral immune surveillance system is that it is prominent in naturally classical MHC class Ia-deficient tadpoles, whereas it is a component in class Ia and conventional T cell competent adults. Nevertheless, while this antiviral system is central for tadpole survival at early stage of FV3 infection, it is also critical for a timely control of viral burden in adult frogs. To better functionally define this novel antiviral immune surveillance system, we examined the initial conditions of FV3 infection that triggers the iVα6 T cell response by using deficient FV3 recombinants, and we defined the antiviral immune response initiated by iVα6 T cells.

The critical role of iNKT cells in antiviral immunity is well documented in mammals, where their pre-activated or memory phenotypes allow them to serve as early responders (within hours following infection) without requiring expansion as conventional T cells [7,24]. Similarly, in *X. laevis*, we have shown a rapid increase of iVα6-Jα1.43 transcript levels (within 1 dpi) at the site of FV3 infection in the peritoneum and in the main site of viral replication in the kidney, concomitant with a decrease in the spleen. Notably, the recruitment of these iVα6 T cells occurs days before significant proliferation of conventional T cells, which become significant only from 3 dpi onward in the spleen [25]. This strongly suggests that iVα6 T cells are rapidly activated and recruited from the spleen by FV3 infection [13].

Here, we provide further evidence that iVα6 T cell recruitment occurs in direct response to FV3 infection and is not indirectly triggered by inflammation induced by heat-killed bacteria or by a replication-defective ∆64-FV3 recombinant. Furthermore, while both attenuated ∆64-FV3 and heat-killed bacterial stimulation induced an early transcriptional induction of the MHC class I-like XNC10 that restricts iVα6 T cells, these elevated expression levels were only maintained in WT-FV3 infected frogs. This suggests a coordinated role of XNC10 and iVα6 T cells in detecting and eliciting a rapid response against ranavirus pathogens. This coordinate response of XNC10 and iVα6 T cells only induced by a productive viral infection is also consistent with the detection of virally-derived products or ligands. It is unknown whether XNC10 binds ligands, and if so the identity of such putative XNC10 ligands is currently unknown, but nucleotide sequence comparisons and 3D-modeling of the putative ligand binding regions indicate that XNC10 could accommodate lipid ligands in a way reminiscent of CD1d [3,26,27]. Thus, it is tempting to speculate that XNC10 can be upregulated and bind products and ligands from early FV3 replication or release of assembled viruses to activate iVα6 T cell response. In this scenario, infection by ∆64-FV3 would not lead to sufficient production and release of FV3-derived products and ligands to initiate iVα6 T cell recruitment and activation.

Interestingly, the correlation between defective viral replication, blunted XNC10 gene expression response, and impaired iVα6 T cell recruitment was more robust in tadpoles. This may be due to the heavier reliance on iT cells in tadpoles. In absence of an efficient classical MHC-I restricted conventional CD8 T cell response, iVα6 T cells are likely to be more directly and critically involved in tadpole host response against FV3. This is evidenced by marked vulnerability of XNC10-deficient transgenic tadpoles lacking iVα6 T cell to FV3 infection, especially at early stages of infection [10]. Our findings using XNC10 tetramer treatment substantiate this initial requirement of iVα6 T cell response for controlling viral infection and host resistance to FV3. Indeed, a partial and transient depletion of iVα6 T cells prompted by injection of XNC10 tetramers shortly before and after infection impairs tadpole immune defenses, which not only leads to more severe viral burden but also decreased survival rate.

While the effector functions of iVα6 T cells are likely to be multifaceted, our previous findings support a role of these cells as immune modulators that influence the polarization of peritoneal macrophages into a more robust anti-viral state [13]. Although XNC10 gene expression is detectable on peritoneal macrophages, in absence of XNC10-specific antibodies, it is currently unknown whether all macrophages express XNC10 molecules at the cell surface and whether macrophages can modulate XNC10 surface expression upon FV3 infection. It is noteworthy that the expression of type I and III IFN, as well as cytokines, such as CSF-1 and IL-18, are transiently altered in parallel to iVα6 T cell depletion. Little is known about the iVα6 T cell mechanism of action, but previous findings have hinted at a modulation of macrophages [10,13]. While type I and III IFN is likely produced and released by many cell types, the rapid increase of their transcript levels in the peritoneum and kidneys upon FV3 infection coincides with the recruitment of monocytic phagocytes, both in tadpoles and adult frogs [18,28,29]. Infiltration of MHC class II+ leukocytes in kidneys at early stages of FV3 infection is also consistent with infiltration of macrophages [25]. CSF-1 and IL-34 are two key macrophage growth factors acting through the same CSF-1 receptor across jawed vertebrates [30]. In *X. laevis*, CSF-1 is required for the commitment and maturation of mature macrophage lineage cells [20], whereas IL-34 stimulates a stronger antiviral effector function by macrophages both in tadpoles and adult frogs [19,31]. The acute decrease in type I and III IFN, as well as CSF-1 and IL-34 at 2 dpi concomitantly with the XNC10 tetramer-mediated iVα6 T cell defect, reinforces the postulated functional link between iVα6 T cells and macrophages. This, of course, does not preclude a possible interaction of iVα6 T cells with other immune cell effectors, as well as a reciprocal effect of macrophages on iVα6 T cells. Indeed, macrophages in mammals are known to be important producers of IL-12 and IL-18, two cytokines that stimulate iNKT cells and promote Th1 adaptive immune response [21,22,32]. The transitory blunted expression of IL-18, and to a lesser extent IL-12, that accompanied iVα6 T cell transient defect, besides being consistent with decreased macrophage function, may have amplified XNC10 tetramer-mediated iVα6 T cell impairment. In any case, it is remarkable that the brief interference of iVα6 T cell function has a long-lasting negative impact on the tadpole ability to control FV3 infection, and ultimately to survive infection.

## Supplementary Materials

The following are available online at https://www.mdpi.com/1999-4915/11/4/330/s1, Figure S1: Viral loads in WT- or ∆64-FV3 infected adult frogs determined by plaque assay. Figure S2: Viral loads in WT- or ∆64-FV3 infected tadpoles determined by plaque assay. Figure S3: Effects of iVa6T cell depletion on XNC10 transcript levels. Table S1: List of qPCR primer sequences. Table S2: FV3 genome copy number in post-mortem tadpoles treated or not with XNC10-tetramers and infected with FV3.
