# Supplementary material for: Critical Role of an MHC Class I-Like/Innate-Like T Cell Immune Surveillance System in Host Defense against Ranavirus (Frog Virus 3) Infection
Source: Viruses. 2019 Apr 6;11(4):330. doi: 10.3390/v11040330 (PMC6521289; doi:10.3390/v11040330)
Supplement: Supplementary file 1 [file viruses-11-00330-s001.pdf]

Table S1: List of qPCR primer sequences

PRIMER	SEQUENCE (5'- 3')
CSF-1	F: ATCGAACTCTGTCCAAGCTGGATG
	R: GGACGAAGCAAGCATCTGCCTTAT
FV3 DNA polymerase II	F: ACGAGCCCGACGAAGACTACA
	R: TGGTGGTCCTCAGCATCC T
GAPDH	F: GACATCAAGGCCGCCATTAAGACT
	R: AGATGGAGGAGTGAGTGTCACCAT
IL -12b	F: GAAACCTGCTGTCCTTCTCTT
	R: TCATGGCAGTTCACCTCATATT
IL-18	F: TGAATGCATATTCTACCAGAGGAC
	R: TGTCTCTTCACAAGCCAGATAAA
IL-34	F: AGCTCTTCTACGTGTATTCCTTGG
	R: TGATAAGCGATTGACCTACCTGGG
TNF-α	F: TGTCAGGCAGGAAAGAAGCA
	R: CAGCAGAGCAAAGAGGATGGT
Type I IFN	F: GCTGCTCCTGCTCAGTCTCA
	R: GAAAGCCTTCAGGATCTGTGTGT
Type II IFN (γ)	F: CTGAGGAAATACTTTAACTCCATTGACC
	R: TTGTAACATCTCCCACCTGTATTGTC
Type III IFN (λ)	F: TCCCTCCCAACAGCTCATG
	R: CCGACACACTGAGCGGAAA
Vα6-Jα1.43 iTCR	F: GGAATTGCTGGTTAATAATTTGGGA
	R: AATGTGAGTTTGTGGAACCCCC
XNC10	F: CTCCATCGCATTCGTCTTTC
	R: TCTTCAACACCAGTCTTGTTT

F: Forward; R: Reverse

Table S2: FV3 genome copy number in post-mortem tadpoles treated or not with XNC10-tetramers and infected with FV3.

	FV3 genome copy number	
**Treatment**	**6-20 dpi [x10^6]**	**30 dpi [x10^3]**
FV3 + APBS (n=15)	1.17 ± 0.498	1.826 ±0.975
FV3 + XNC10-T (n=15)	7.28 ± 2.26 0.0501[a]	0.944 ±0.498 P: 0.5571[b]

Three week-old, stage 55 tadpoles were i.p. injected with APBS or XNC10-T prior and after FV3 infection. Survival was monitored for 30 days, during which dead tadpoles were collected to determine post-mortem viral loads. At 30 dpi, the remaining survivors were euthanized. FV3 genome copy number was determined by qPCR using primers for FV3 DNA polymerase II on genomic DNA extracted from whole tadpoles.

Note: [a]P value of viral loads from 6-20 dpi FV3 + APBS compare to 6-20 dpi FV3 + XNC10-T. [b]P value of viral loads from euthanized tadpoles at 30 dpi FV3 + APBS compare to 30 dpi FV3 + XNC10-T

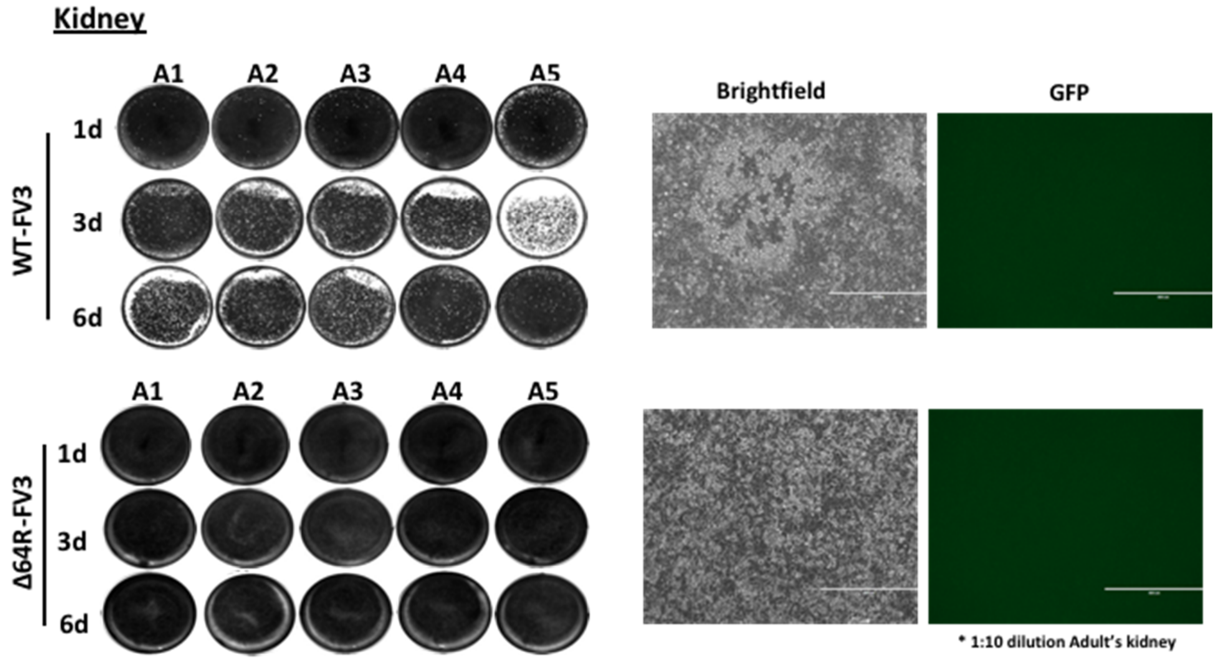

Fig. S1: Viral loads in WT- or Δ64-FV3 infected adult frogs determined by plaque assay. FV3 infectious particles recovered from two year-old adult kidneys (5 individuals per group), i.p. infected with $1x10^6$ PFUs of either WT- or Δ64-FV3 for 1, 3, and 6 days, determined by plaques assay on BHK-21 cells. Left panel: Plates showing plaque accumulation with WT-FV3 but not Δ64-FV3. Right panel: Magnification showing WT-FV3 plaques on bright field microscope and absence of Δ64-FV3 plaques on fluorescent microscope.

Kidney

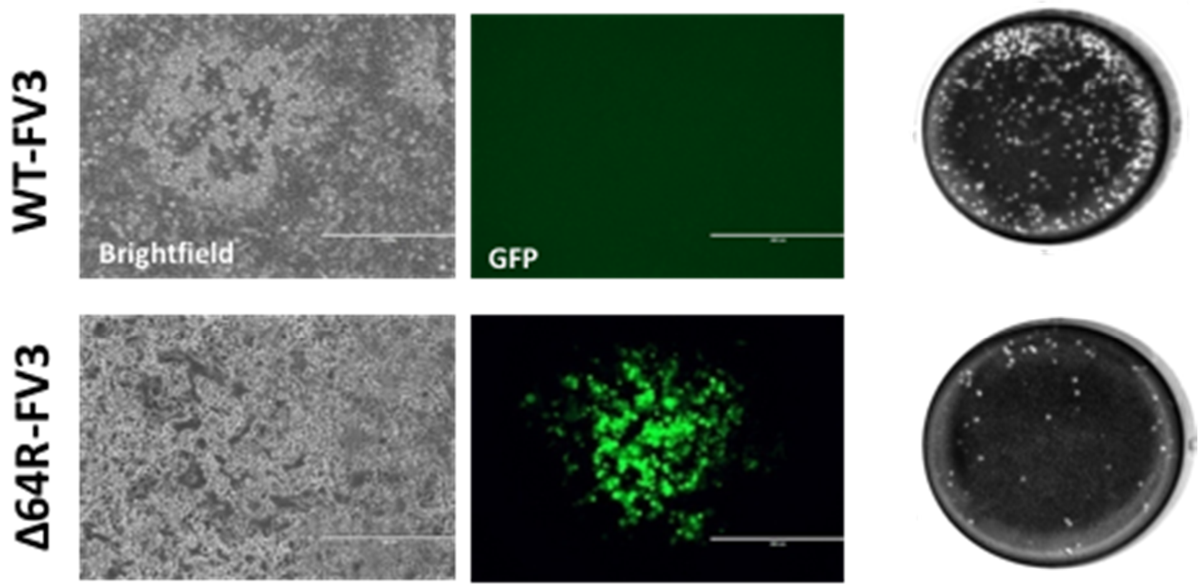

Fig. S2: Viral loads in WT- or Δ64-FV3 infected tadpoles determined by plaque assay. FV3 infectious particles recovered from three week-old (stage 55) tadpoles (5 individuals per group), i.p. infected with $1x10^4$ PFUs of either WT- or Δ64-FV3 for 6 days, determined by plaques assay. Left panel: Phase contrast (left) and fluorescence (center and low magnification (right) microscopy showing plaques. All plaques produced by Δ64R-FV3 were EGFP-positive, indicating that they were not contaminated with the WT-FV3 virus.

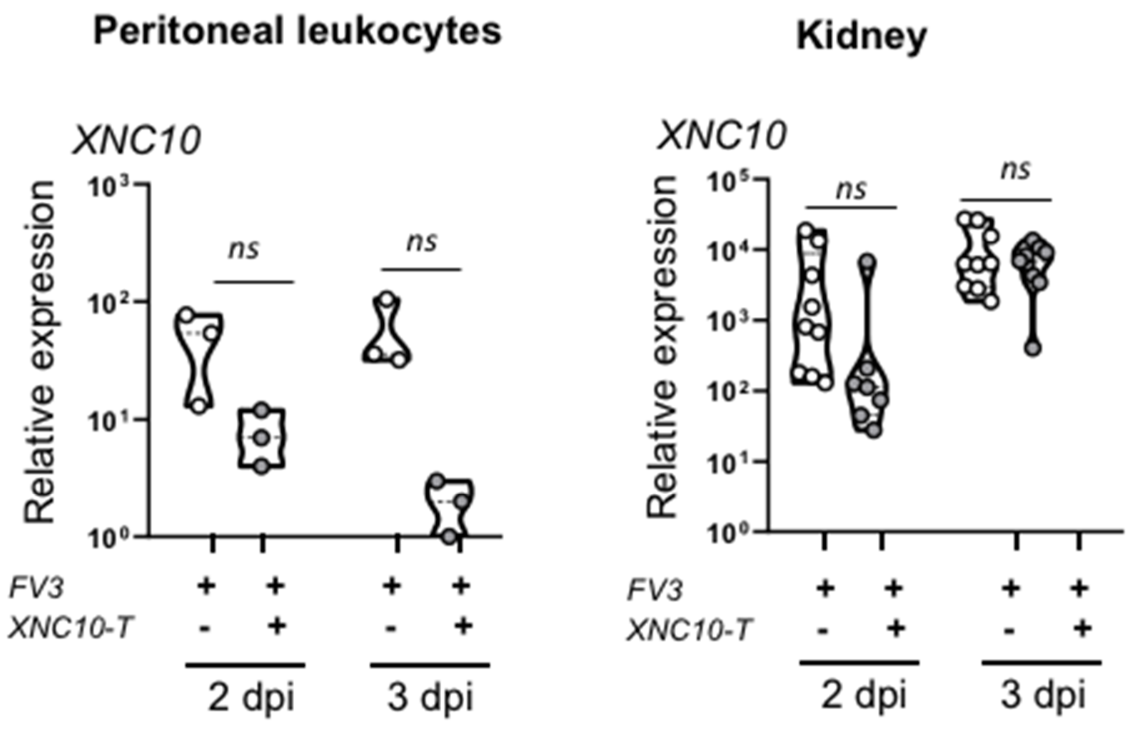

Fig. S3: Effects of iVa6T cell depletion on XNC10 transcript levels. PLs and kidneys were collected from three week-old (stage 55) tadpoles that had been injected with 1 μg XNC10 tetramers or vehicle control, 1 day pre- and 1 day post- i.p injection with 10,000 PFUs of FV3, at the indicated time points (n = 9). Gene expression of XNC10 in peritoneal leucocytes (**A**) and kidneys (**B**) is shown. Results are normalized to an endogenous control and presented as relative expression compared with the lowest observed value, according to the ΔΔCt method. For peritoneal leucocytes, each dot represents a pool of 3 tadpoles, while for kidneys, each dot represents a single tadpole; * p<0,05 denotes statistically significant differences between the indicated groups (one way ANOVA followed by Tukey`s multiple comparison test).
